# The Biochemical Profile of Post-Mortem Brain from People Who Suffered from Epilepsy Reveals Novel Insights into the Etiopathogenesis of the Disease

**DOI:** 10.3390/metabo10060261

**Published:** 2020-06-23

**Authors:** Ashna M. Lalwani, Ali Yilmaz, Halil Bisgin, Zafer Ugur, Sumeyya Akyol, Stewart Francis Graham

**Affiliations:** 1 Department of Biochemistry and Molecular Biology, Hamilton College, 198 College Hill Rd, Clinton, NY 13323, USA; alalwani@umich.edu; 2 Department of Computational Medicine and Bioinformatics, University of Michigan, Ann Arbor, MI 48109, USA; 3 Department of Obstetrics and Gynecology, Beaumont Health System, 3601 W. 13 Mile Road, Royal Oak, MI 48073, USA; ali.yilmaz@beaumont.org (A.Y.); zaferugur34@gmail.com (Z.U.); 4 Oakland University-William Beaumont School of Medicine, 586 Pioneer Dr, Rochester, MI 48309, USA; 5 Beaumont Research Institute, Beaumont Health, 3811 W. 13 Mile Road, Royal Oak, MI 48073, USA; Sumeyya.Akyol@beaumont.org; 6 Department of Computer Science, Engineering, and Physics, University of Michigan-Flint, 303 E. Kearsley St, Flint, MI 48502, USA; bisgin@umich.edu

**Keywords:** epilepsy not-otherwise-specified (ENOS), metabolomics, proton nuclear magnetic resonance (^1^H-NMR), targeted mass spectrometry, metabolic pathways

## Abstract

Epilepsy not-otherwise-specified (ENOS) is one of the most common causes of chronic disorders impacting human health, with complex multifactorial etiology and clinical presentation. Understanding the metabolic processes associated with the disorder may aid in the discovery of preventive and therapeutic measures. Post-mortem brain samples were harvested from the frontal cortex (BA8/46) of people diagnosed with ENOS cases (*n* = 15) and age- and sex-matched control subjects (*n* = 15). We employed a targeted metabolomics approach using a combination of proton nuclear magnetic resonance (^1^H-NMR) and direct injection/liquid chromatography tandem mass spectrometry (DI/LC-MS/MS). We accurately identified and quantified 72 metabolites using ^1^H-NMR and 159 using DI/LC-MS/MS. Among the 212 detected metabolites, 14 showed significant concentration changes between ENOS cases and controls (*p* < 0.05; *q* < 0.05). Of these, adenosine monophosphate and *O*-acetylcholine were the most commonly selected metabolites used to develop predictive models capable of discriminating between ENOS and unaffected controls. Metabolomic set enrichment analysis identified ethanol degradation, butyrate metabolism and the mitochondrial beta-oxidation of fatty acids as the top three significantly perturbed metabolic pathways. We report, for the first time, the metabolomic profiling of postmortem brain tissue form patients who died from epilepsy. These findings can potentially expand upon the complex etiopathogenesis and help identify key predictive biomarkers of ENOS.

## 1. Introduction

Epilepsy not-otherwise-specified (ENOS) is one of the most common chronic disorders impacting human health, and the third most common serious neurological disorder, following stroke and Alzheimer’s disease [1,2]. In 2015, 1.2% of the total US population had active epilepsy. This equates to 3.4 million people with epilepsy nationwide: 3 million adults and 470,000 children equaling ~50 million people worldwide [3]. It can affect any person (affecting males and females equally), at any age and present, with varying levels of severity [4,5,6]. Studies have indicated that genetics plays a strong role in epilepsy susceptibility, as shown by the elevated concordance rates among twins, specifically among identical versus fraternal twins [7,8,9]. Investigators have reported that among identical twins, if one has epilepsy, then the other has a 50–91% chance of also developing the disorder. In non-identical twins, if one twin has epilepsy, the other twin has a 0–33% chance of developing the disease [10].

Presently, the most effective therapeutic interventions available for epilepsy are anti-epileptic drugs (AEDs). However, AEDs are highly impactful, have adverse effects and are ineffective in large populations of those people who suffer from epilepsy [11,12]. Due to the unknown multifactorial etiology and the necessity for early intervention in ENOS, current research focuses on the early detection [13,14,15,16] and prevention of ENOS [17,18,19], as well as increasing our understanding of the etiopathogenesis of the disease [20,21,22].

Metabolomics, or metabolic profiling, is one of the younger “omics” technologies which comprehensively studies metabolic pathways in biological systems with the focus on metabolites [23,24]. Metabolomics encompasses high-throughput identification and elucidation of the small molecule metabolites that are produced by cells, tissues and microorganisms [25,26,27]. Metabolomics has previously been employed in attempts to diagnose epilepsy, however no single peripheral biomarker panel has demonstrated proven effectiveness [28,29,30,31]. A study in 2018 used biofluids, specifically microRNAs in blood, to look at the dysregulation of epileptic brain tissue. However, as there is a limited mechanistic understanding of miRNAs, the implementation of relevant biomarkers cannot currently prevent epileptogenesis or alter treatment [32]. In addition, a recent study by Raoof et al. (2018) report a dual-center, dual-platform approach, demonstrating the biomarker potential of circulating miRNAs for diagnosing epilepsy, while providing some mechanistic insights into the underlying pathological mechanisms [33].

In this present study, and for the first time, we combine ^1^H nuclear magnetic resonance (^1^H-NMR) and direct injection liquid chromatography, coupled with mass spectrometry (DI/LC-MS/MS), to quantitatively profile postmortem human brain tissue from epileptic patients and compared them to age- and gender-matched controls. We believe that this approach will help us to identify the central biomarkers of ENOS, while uncovering previously unreported biochemical pathways associated with the disease.

## 2. Results

Using ^1^H-NMR and DI/LC-MS/MS, we biochemically profiled post-mortem human brain tissue from people who died from ENOS and compared them with age- and gender-matched controls. We accurately identified and quantified 72 metabolites using ^1^H-NMR and 159 using DI/LC-MS/MS. Due to the complementarity between the two techniques, there was a certain degree of observed overlap in terms of the metabolites measured (19 metabolites). The concentration difference between the two platforms was <10% coefficient of variation (CV), which is considered to be within the acceptable range [34]. To account for the two measurements, we took the average value for the individual metabolite and used this concentration value in our analyses, leaving us with 212 metabolites. In order to detect any intrinsic variation and identify potential outliers, principal component analysis (PCA) was performed on the data and outliers were highlighted as being outside the 95% confidence interval (*p* < 0.05; Hotelling’s T^2^).

The score plots of each PCA model showed no outlier for each class (Supplementary Figure S1). Table 1 reports the results of univariate comparisons of important demographic factors, such as age and gender (*p* > 0.05).

Using the metabolite concentrations, pair-wise univariate, and multi-variate statistical comparisons were carried out between controls and ENOS cases. A univariate analysis of the data revealed that, of the 212 metabolites, 14 of them were at statistically and, significantly different concentrations between ENOS and control tissue (Supplementary Table S1; *p* < 0.05; *q* < 0.05).

**Figure 1:**
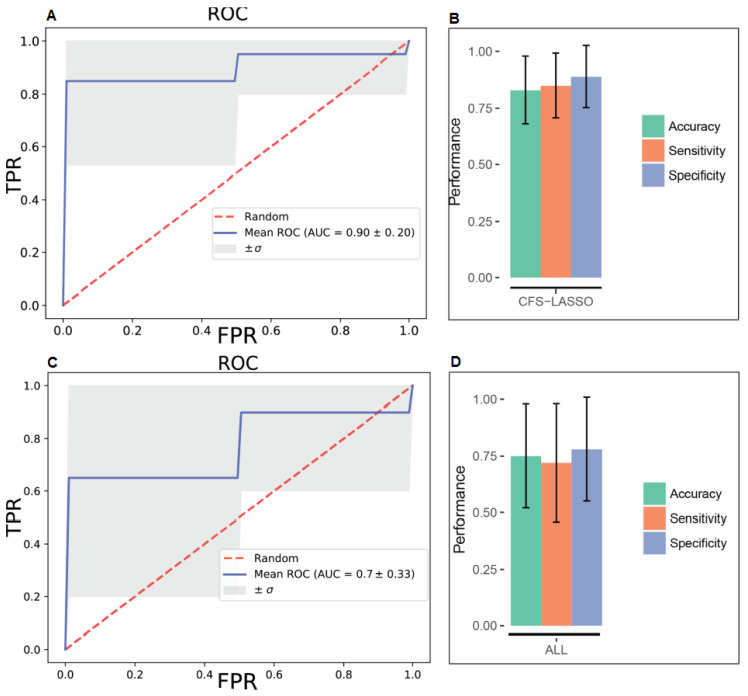
Receiver operating characteristic (ROC) curves for support vector machine (SVM) models built on all metabolite concentration values and the top five metabolites selected by both CFS and LASSO variable selection algorithms, respectively (**A** and **C**) and corresponding performance metrics (**B** and **D**). FPR—false positive rate; TPR—true positive rate.

Following outlier detection, and performing a univariate analysis of the data, we developed diagnostic models using various statistical algorithms. In particular, we developed two support vector machine (SVM) and two logistic regression (LR)-based diagnostic models using the concentrations of (i) all recorded metabolites and (ii) the top five common metabolites, as selected by both Correlation based feature selection (CFS) and least absolute shrinkage and selection operator (LASSO) algorithms. Supplementary Table S2 demonstrates that in both cases (all metabolites vs. only top five metabolites), statistical models developed using the SVM approach outperformed its LR-based counterpart in discriminating ENOS cases from the control group. Combining all the metabolite measurements with SVM optimized parameters, we developed a model with an AUC (area under curve) = 0.77, with 75% accuracy (Figure 1A,B). While this model was statistically significant (*p* < 0.001; Supplementary Figure S2), the model developed using the concentrations of the top-five metabolites (selected using both algorithms) had an AUC = 0.90, with 83% accuracy. In Figure 1C,D, we present all the model metrics, including sensitivity and specificity, showing that the top-five metabolite model performed best on all fronts to include observed and predicted group membership (Supplementary Table S2 and Supplementary Figure S3).

**Figure 2:**
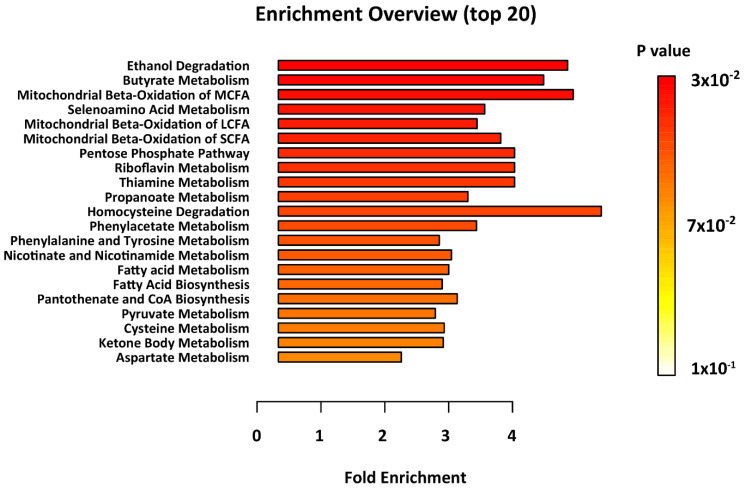
Results of the metabolite set enrichment analysis. Metabolic pathways with *p* < 0.05 were considered to be significantly perturbed.

Metabolomics enrichment analysis highlighted thirteen metabolic pathways as significantly disturbed in epileptic brain, as compared with controls. These include; ethanol degradation, butyrate metabolism, mitochondrial beta-oxidation of medium-, long- and short-chain fatty acid (MCFAs, LCFAs, SCFAs) metabolism, and fatty acid biosynthesis (Figure 2, Supplementary Table S3).

## 3. Discussion

**Figure 3:**
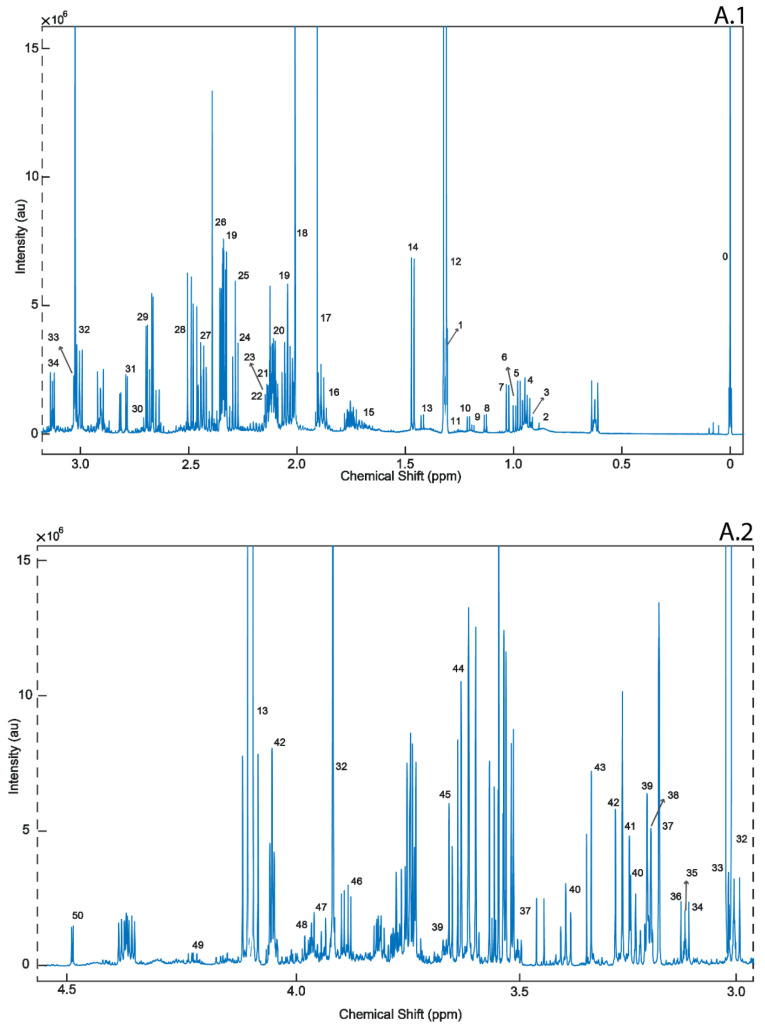

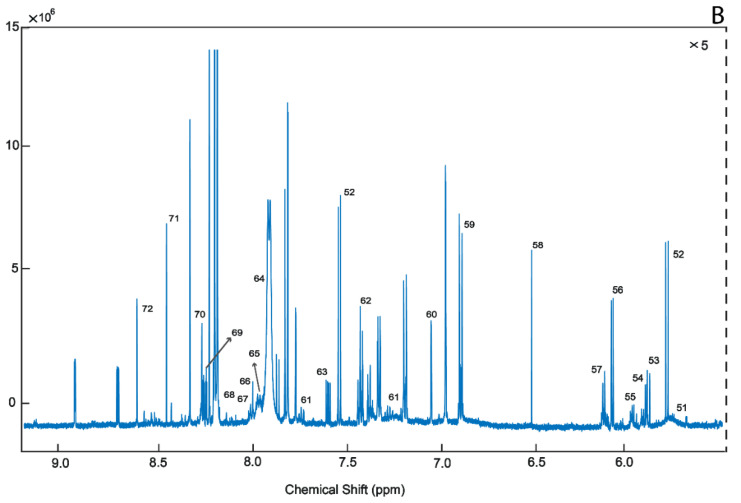
A representative 1D 1H-NMR of PM brain harvested from the BA8/46 region from an ENOS sufferer. Aliphatic region covering (0 to 3.0 ppm: **A.1** and 3.0 to 5.00 ppm: **A.2**), and five-fold scaled aromatic region (**B**), respectively. 0. Sodium 2,2-dimethyl-2-silapentane-5-sulfonate (DSS) 1. Threonine, 2. 2-Hydroxyisovalerate, 3. Pantothenate, 4. Leucine, 5. Isoleucine, 6. Valine, 7. 3-Hydroxyisobutyrate, 8. Isobutyrate, 9. Propylene glycol, 10. Isopropanol, 11. 3-Hydroxybutyrate, 12. 3-Hydroxyisovalerate, 13. Lactate, 14. Alanine, 15. Lysine, 16. gamma-Aminobutyric acid (GABA), 17. Acetate, 18. *N*-Acetylaspartate, 19. Glutamate, 20. Glutamine 21. Methionine, 22. *O*-acetylcarnitine, 23. Acetone, 24. Acetoacetate, 25. 4-Aminobutyrate, 26. Pyruvate, 27. Succinate, 28. Pyroglutamate, 29. Citrate, 30. Dimethyl amine, 31. Aspartate, 32. Creatine, 33. Creatinine, 34. Malonate 35. Ethanolamine, 36. Dimethyl sulfone, 37. Choline, 38. Carnitine, 39. *O*-phosphocholine, 40. Sn-Glycero-3-Phosphocholine, 41. Taurine, 42. Myo-Inositol, 43. Methanol, 44. Glycine, 45. Glycerol, 46. Ascorbate, 47. Anserine, 48. Serine, 49. Inosine, 50. Glucose, 51. Urea, 52. Uracil, 53. Uridine diphosphate glucose, 54. Guanosine triphosphate, 55. UDP-Galactose, 56. Inosine, 57. Adenosine triphosphate, 58. Fumarate, 59.Tyrosine, 60. π-Methyl histidine, 61. Tryptophan, 62. Phenylalanine, 63. Niacinamide, 64. Adenine, 65. Adenosine monophosphate, 66. Adenosine Diphosphate, 67. ATP, 68. Pantothenate, 69. Hypoxanthine, 70. Histamine, 71. Formate, 72. Inosine monophosphate.

To our knowledge, this is the first study employing ^1^H-NMR and DI/LC-MS/MS to quantitatively profile postmortem epileptic human brain harvested for the frontal cortex (BA8/46) and compared them with age- and gender-matched controls. Specifically, Brodmann Region 8/46 is part of the dorsolateral prefrontal cortex (DLPFC), reported to be highly involved with executive function [35]. The DLPFC is also responsible for short-term memory retention, sensory input and motor signaling, that combine in an integrated response, all of which are altered in epileptic seizures [36]. Schizophrenia, depression and stress are also linked to the malfunction of Brodmann Region 8/46 [37]. Of the 212 recorded metabolites, we identified only 14 to be at statistically significantly different concentrations (*p* < 0.05; *q* < 0.05), when the postmortem brains of ENOS sufferers were compared with controls. A representative 1D ^1^H-NMR of PM brain harvested from the BA8/46 region from an ENOS sufferer allowed us to confidently quantify all metabolites profiled by NMR (Figure 3). On further inspection of the individual class data, we found that none of the samples in the control or ENOS group could be considered as outliers using PCA analysis (Supplementary Figure S1).

The use of all the metabolite recordings with SVM and LR successfully classified epileptic patients, with an average accuracy rate of 0.78 and 0.75, respectively. This not only showed the potential of metabolite measurements in such a diagnosis task, but also warranted further research on the most useful panels, which could eventually serve as biomarkers. To discover a narrowed subset of metabolites which is more feasible for diagnosis of ENOS, we employed both CFS and LASSO feature selection algorithms, to take advantage of multiple methods for a presumably more informative and robust set of features.

Given that every feature selection algorithm adopts a different approach and a subjective function, which may change priorities, it is not unusual to observe disagreements in the resulting features and their order of importance. In our case, of the top six metabolites, five of them were found to be the same, with order differences by two feature selection algorithms, which may be considered as a consensus. Then, we carried out our modeling with the common set of metabolites, which both CFS and LASSO methods agreed on, i.e., AMP, O-Acetylcholine, l-Fucose, Isobutyric acid, and Glycerol. We built SVM and LR models, which were evaluated through 10-fold CV again to test their capacity for a better or equivalent diagnostic accuracy. Our experiments with this narrowed set of features gave a better average diagnostic accuracy (0.83 vs. 0.78 and 0.75 vs. 0.80). Similarly, we observed a better performance in terms of AUC (0.90 vs. 0.77 and 0.79 vs. 0.70), sensitivity (0.85 vs. 0.67 and 0.87 vs. 0.80), and specificity (0.89 vs. 0.65 and 0.73 vs. 0.67) for the smaller set of features, which might be the most representative metabolites amongst others. While higher sensitivity and specificity values indicated more accurate decisions for both epileptic and non-epileptic groups, lower standard deviations also suggested consistent predictions over different training and testing groups with the same set of features. In a recent study, Wu et al. (2016) investigated metabolic and genomic signatures as potential non-invasive biomarkers for epilepsy [38]. In said study, a highly consistent predictive metabolite logistic regression model with reduced lactate and increased creatine plus phosphocreatine (Cr + PCr) and choline provided AUC, sensitivity specificity values of 0.88, 0.85 and 0.79, respectively. In our study, we go one step further; we profiled brain tissues from ENOS and produced powerful models with an AUC = 0.90, with sensitivity = 0.85 and specificity = 0.83 (Supplementary Figure S2). To the authors’ knowledge, these are the highest diagnostic values to be reported in the literature.

The results of the MSEA highlighted oxidative metabolism of lipids as significantly perturbed in epileptic samples (*p* < 0.05; *q* < 0.05). It has long been hypothesized that alterations in lipid metabolism contribute to neurodegenerative disease. Butyrate metabolism was the major biochemical pathway found to be significantly perturbed in the postmortem epileptic brain. Butyrate metabolism is essential for acetyl-CoA biosynthesis and therefore directly involved in lipid synthesis and the oxidation of the tricarboxylic acid (TCA) cycle. Acetyl-CoA levels (in tandem with the carnitine system) heavily impact mitochondrial beta oxidation, which metabolize lipids (MCFAs, SCFAs, and LCFAs) for energy production [39]. Therefore, the perturbation of butyrate metabolism may lead to disrupted energy homeostasis through the lack of ATP. MCFAs are of particular importance, as they serve several biological functions within the brain, and as agonists of the peroxisome proliferator-activated receptors. They do not require proteins to be metabolized, and therefore serve as (1) energy processing fuel under pathological conditions, such as acute inflammation, and (2) enhancers of insulin sensitivity of tissues [39,40]. SCFAs, comparatively, play a stronger role in immune homeostasis [41]. The activation of SCFAs occurs in the liver and other tissues by acyl CoA synthetase leading to AceCS2. Other studies have shown the critical link between the mitochondrial beta oxidation of SCFAs and ATP: when lacking AceCS2, ATP content declined by 50% in AceCS2 mice versus negative controls [42]. Both MCFAs and SCFAs contribute to intracellular signaling and regulation of cell metabolism, as well as the control of cell death and survival.

Epileptic individuals characteristically experience neural hypoxic events, or cell death, causing seizures. Two of the major metabolic pathways identified as being significantly perturbed in postmortem epileptic brain (butyrate metabolism and ethanol degradation) rely heavily on oxidation. Oxygen consumption using mitochondrial beta-oxidation is typically accompanied with the generation of reactive oxygen species (ROS) and the excessive accumulation of MCFAs is connected to respiratory chain complex impairment [43]. Complex 1 (C1) of mitochondrial beta-oxidation is the biochemical hallmark of seizure induced neuronal cell death and is the most susceptible to oxidative stress. As a major source of superoxide anions, C1 is a target of ROS production and redox signaling, causing reduced electron transport chain efficiency and therefore a further reduction of ATP [44]. When superoxide levels are elevated, mitochondrial respiratory chain dysfunction (MRCD) initiates the apoptosis compound cascade, causing neural apoptosis, and therefore seizure activity. Alternatively, MRCD decreases neuroprotective strategies, causing further MRCD which increases endonuclease G, AiF (apoptosis inducing factor) and Smac (second mitochondria-derived activator of caspases). These compounds all contribute to the initiation of the apoptosis cascade, again leading to seizure activity by cell death [45]. This feedback loop is confirmed by prolonged seizure activity that causes increased oxidative stress and MRCD. The transient opening of nonspecific mitochondrial inner membrane pores under such cellular stress results in the collapse of several transmembrane potential releasing apoptotic compounds, including cytochrome C and Bcl2. Furthermore, the link between oxygen deprivation and seizure activity is corroborated by several categorically oxygen-depriving disorders, diseases and injuries (stroke, traumatic brain injury, leading to epileptic seizures [46]. Consequently, the disruption of butyrate metabolism, in combination with the mitochondrial beta-oxidation of fatty acids and ethanol degradation, may explain neuronal death and seizure activity attributed to oxidative malfunction.

**Figure 4:**
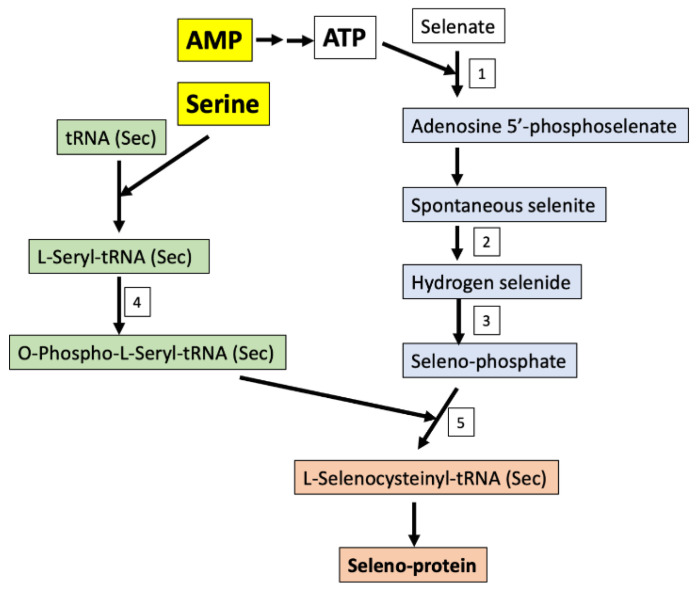
Seleno-protein synthesis in humans using organic or inorganic sources of selenium from the diet. Enzymes: 1-Sulfate adenylyltransferase; 2-NADPH (reduced nicotinamide adenine dinucleotide phosphate)-thioredoxin reductase; 3-Selenophosphate synthase; 4-*O*-phosphoseryl-tRNA(Sec) kinase; 5-l-selenocysteinyl-tRNA(Sec) synthase and *O*-phosphoseryl-tRNA: Selenocysteinyl-tRNA synthase.

The perturbation of seleno-amino acid metabolism is also considered very important in epilepsy. The importance of seleno-proteins in human health is reflected in those patients with inborn errors in seleno-proteins, or their biosynthetic factors that show genetic generalized epilepsy in the case of mutations to related genes [47]. As an essential trace element, selenium normally substitutes for sulfur in sulfur containing amino acids and creates seleno-amino acids without changing said protein’s current function and structure, but contributes to a greater antioxidant and redox-protective potential [48]. The only seleno-amino acid that can be synthesized in higher animals by using inorganic (selenite and selenate) and/or organic (selenocysteine (Sec) and selenomethionine (SeMet)) selenium sources is Sec. The de novo synthesis of Sec is always achieved by a phosphorylated intermediate in a tRNA-dependent reaction. The initial reactions of the synthetic pathway include serine and AMP. Selenate is added to AMP to produce adenosine 5′-phosphoselenate culminating in seleno-phosphate after numerous intermediary steps. Contrastingly, l-serine is combined with tRNA to produce *O*-phospho-l-seryl-tRNA. These two metabolic pathways combine to produce l-seleno-cysteinyl-tRNA, used for seleno-protein synthesis (Figure 4). During seleno-protein synthesis, Sec is inserted as the 21st amino acid in the protein structure. While the fundamental functions are not yet known, only 25 Sec-containing proteins have been identified in the human body [49]. Interestingly, glutathione peroxidase (GPX), an antioxidant enzyme that catalyzes the conversion of hydrogen peroxide to water and hydroperoxides to less toxic molecules, has been associated with epileptic seizures. Eight members of GPX have been identified to include GPX4, which functions as a phospholipid hydroperoxidase (reduces lipid peroxides which are known to be toxic). This enzyme is also reported to have a crucial function during embryogenesis (REF). A recent study demonstrated the role of Sec in GPX4; the mutation that led to the exchange of the amino acid Sec with Cys in the GPX4 enzyme structure (*GPX4^cys/cys^* mice), causing general dysfunction in a variety of cells and tissues, manifesting in death by epileptic seizures [50]. The mice in this study all died three weeks following birth. It is hypothesized that GPX4 regulates ferroptosis, a relatively new term to define iron- and lipid-dependent regulated cell death associated with GSH depletion and the production of lipid peroxides by lipoxygenase enzymes. Increased lipid peroxidation in postmortem brain from epilepsy patients further suggests that ferroptosis represents a cellular mechanism underlying excitotoxic neuronal injury [51]. As epilepsy is commonly associated with mitochondrial disease, targeting GPX4 to stimulate the enzymatic removal of lipid hydroperoxides could present a potential interventional strategy for mitochondrial disease-associated epilepsy, such as pontocerebellar hypoplasia type 6 [52].

Univariate analyses highlighted several metabolites to be at significantly different concentrations between epileptic brain and control extracts. These include: AMP, 3-hydroxyisovalerate, *O*-Phosphocholine, C6 C4:1-DC or hexanoylcarnitine (fumarylcarnitine), and C4 butyrylcarnitine. AMP is believed to be at lower concentrations in mitochondria, suffering from dysfunctional β-oxidation, which corroborates with other hypotheses that epileptogenesis is caused by dysfunction of the oxidative system [53]. The lack of ATP leads to a chain dysregulation which subsequently results in apoptosis and therefore cell death in the brain, causing epileptic fits [54].

Moreover, 3-hydroxyisovalerate was found to be significantly higher in epileptic patients versus the controls. Notably, 3-hydroxyiolvalerate is a precursor to acetyl CoA, which is key in the activation of the metabolism of fatty acids. The lack of 3-hydroxyisovalerate metabolism indicates dysfunction in the regulation of fatty acid metabolism as well as energy homeostasis [55]. The outcome being lower levels of ATP, leading to a lack of energy homeostasis within the brain and consequent neuronal cell death causing seizure activity. Possible secondary metabolomic links include ACeSC2 and complex1 of the metabolomic system specifically, as previously described.

*O*-Phosphocholine was comparatively lower in epileptic patients versus healthy control brains. It is one of the more commonly dysregulated metabolites in neurological disorders and diseases [56]. As an intermediate of phosphatidylcholine, it is critical for the suppression of the immune response [57]. Its ability to increase acetylcholine directly impacts memory and bodily functions. *O*-phosphocholine is also heavily indicated in the breakdown of fat and fatty acids, specifically in the liver. Furthermore, it potentially plays a protective role in the intestines within the digestive system. It is also suggested to be a β-cell biomarker, as it is related to beta β-cell apoptosis and dysfunction [58]. When considering amino acid metabolism, this compound can be used to make glycine and serine by different synthetic metabolic pathways. If phosphocholine cannot be converted to choline, then there is a glycolysis switch, again leading to low ATP and oxidative malfunction.

C6 C4:1-DC, or hexanoylcarnitine, an acetyl-l-carnitine, was found to be at significantly lower concentrations in epileptic patients versus controls. This compound is implicated in the transport of LCFAs during mitochondrial beta oxidation. l-carnitine and acetyl-l-carnitine also play neuroprotective roles in the developing brain. This compound has been prescribed to Alzheimer’s disease patients, as it has been linked to diminished memory loss, depression and liver-function related brain issues [59]. It is similar to l-carnitine, in that it increases ambulatory activity and elevates carnitine levels in the blood and brain, but it is not effective in decreasing oxidative damage [39]. In rat brains, it decreases malondialdehyde (MDA), oxo8dg/oxo8G, and nitrotyrosine [60]. It is also a neuroprotective compound against ischemia and other key mitochondrial metabolic roles. It can improve energy in the system, decrease oxidative stress and prevent subsequent cell death in brain injuries. When not present in a brain injury situation, there is an increase of oxidative stress, a lack of energy homeostasis and increased cell death [61].

C4 or butyrylcarnitine was also identified to be at significantly lower concentrations in the epileptic brain when compared with the controls. As an acylcarnitine, this compound has been identified as a potential biomarker in diagnosing patients with neuromuscular phenotype [62]. As such, it is a substantiated potential biomarker of muscle distress and cell death, correlated with epileptogenesis. C4 is deficient in people with short-chain acyl-CoA dehydrogenase (SCAD) deficiency. This disorder is characteristic in infants with muscle weakness and middle-aged patients with chronic muscle myopathy. The deficiency caused myopathy leads to further beta oxidation defects in cultured fibroblasts, skeletal muscle cells and the fresh muscle of humans. Currently, there is precedent for using C4 in diagnosis of disorders related to beta oxidation dysfunction (as in SCAD patients) [63]. The results of this study seem to be consistent with the previously reported literature using antemortem tissue. For example, in a study by Wang et al., they report high levels of proline and glutamate and low levels of fatty acids in serum, which is consistent with the dysfunction of oxidative metabolism and fatty acid degeneration, leading to the increased inflammatory effect and energy deficiency found in our study [29]. Furthermore, in a study examining urine, the authors confirm our results by implicating the dysfunction of mitochondrial oxidation pathways’ intermediates, such as glutamate, lysine and proline precursors, leading to cell death and neurotoxicity [64]. The record of metabolic dysfunction at the point of injury in epileptic sufferers is both corroborated and expanded upon by this first reported study of ENOS in postmortem brain tissue from Brodmann Area 8/46.

## 4. Materials and Methods

### 4.1. Tissue Samples

A limited number of samples and tissue volume were available for the purposes of this study. Tissue samples from the frontal cortex (BA8/46) were obtained from post-mortem ENOS cases (*n* = 15) and age- and gender-matched control subjects (*n* = 15). Tissues were obtained from the NIH NeuroBioBank (Bethesda, MD, USA). The available clinical and demographic details, such as age, gender and post-mortem delay, can be found in Supplementary Table S4. Frozen tissue samples (~1.5 g) were lyophilized and milled to a fine powder under liquid nitrogen to limit heat production and were stored at −80 °C prior to preparation. This study was approved by the Beaumont Institutional Review Board (IRB# 2020-189).

### 4.2. Sample Preparation

#### 4.2.1. ^1^H-NMR Sample Preparation

For ^1^H-NMR, 50 mg samples were extracted in 50% methanol/water (1 g/mL) in a sterile 2 mL Eppendorf tube. The samples were mixed for 20 min and sonicated for 20 min, and the protein was removed by centrifugation at 13,000× *g* at 4 °C for 30 min. Supernatants were collected, dried under vacuum using a Savant DNA Speedvac (Thermo Scientific, Waltham, MA, USA), and reconstituted in 285 μL of 50 mM potassium phosphate buffer (pH 7.0), 30 μL of Sodium 2,2-dimethyl-2-silapentane-5-sulfonate (DSS) and 35 μL of D_2_O. Then, 200 μL of the reconstituted sample was transferred to a 3 mm Bruker NMR tube for analysis. All samples were housed at 4 °C in a thermostatically controlled SampleJet autosampler (Bruker-Biospin, Billerica, MA, USA) and heated to room temperature over 3 min prior to analysis by NMR [65].

#### 4.2.2. DI/LC-MS/MS Sample Preparation

Notably, 10 mg of powdered PM brain tissue was extracted using 150 µL of extraction solvent (85% ethanol and 15% phosphate-buffered saline solution (PBS)). The samples were ultrasonicated 10 min in ice and vortexed for 1 min. Proteins and other impurities were separated by centrifugation at 13,000× *g* for 15 min at 4 °C. The supernatant was collected and 10 µL was used for analysis by DI/LC-MS/MS [65].

### 4.3. Data Collection and Metabolic Profiling

#### 4.3.1. ^1^H-NMR Analysis

Using a randomized running order, all 1D ^1^H-NMR data were recorded at 300 (± 0.5) K on a Bruker ASCEND HD 600 MHz spectrometer (Bruker-Biospin, Billerica, MA, USA), coupled with a 5 mm TCI cryoprobe. For each sample, 256 transients were collected as 64k data points, with a spectral width of 12 kHz (20 ppm), using a pulse sequence called CPP WaterSupp (Bruker pulse program: pusenoesypr1d), developed by Mercier et al. [66] and an inter-pulse delay of 9.65 s. The data collection protocol included a 180 s temperature equilibration period, fast 3D shimming using the *z*-axis profile of the ^2^H-NMR solvent signal, receiver gain adjustment, and acquisition. The free induction decay signal was zero filled to 128k and exponentially multiplied with a 0.1 Hz line broadening factor. The zero and first order phase constants were manually optimized after Fourier transformation, and a polynomial baseline correction of the FID (degree 5) was applied for precise quantitation. The singlet produced by the DSS methyl groups was used as an internal standard for chemical shift referencing (set to 0 ppm, concentration 1000 µM) and for quantification. All spectra were processed and analyzed using Chenomx NMR Suite (v8.1, Edmonton, AB, Canada) [65].

#### 4.3.2. DI/LC-MS/MS Analysis

Targeted mass spectrometric analysis was carried out using the commercially available Absolute IDQ p180 kit (Biocrates Life Sciences AG, Innsbruk, Austria). Data was acquired using a Xevo TQ-S mass spectrometer coupled to an Acquity I Class UPLC system (Waters Technologies Corporation, Milford, MA, USA), as per the manufacturer’s instructions. The system allows for the accurate quantification of up to 188 endogenous metabolites, including amino acids, acylcarnitines, biogenic amines, glycerophospholipids, sphingolipids, and sugars. Sample registration and an automated calculation of metabolite concentrations and the export of data were carried out with Biocrates MetIDQ software (Biocrates Life Sciences AG, Innsbruk, Austria).

### 4.4. Statistical Analysis

#### 4.4.1. Data Preprocessing

Sum-to-one normalization was initially employed to eliminate the variation due to any potential dilution effect. Subsequently, we applied a z-score normalization protocol [67,68], which mapped all metabolites to the same scale for compatibility.

#### 4.4.2. Univariate Analysis

Metabolite concentrations were analyzed using a Student’s *t*-test or a Wilcoxon signed rank test, based on the results of their parametric test. For metabolites which were measured using both analytical platforms, an average value was used for all additional analyses. False discovery rates (FDR, *q*-values) were also calculated to account for multiple comparisons [69].

#### 4.4.3. Feature Selection

We employed the correlation-based feature selection (CFS) algorithm [70] in WEKA [71] and the LASSO method to eliminate redundancy in the feature space.

#### 4.4.4. Predictive Models with Support Vector Machines

We built our predictive models by using support vector machines (SVMs), which are capable of handling both linear and non-linear data, with a choice of kernel function that maps the data to a higher space where it can be separable. In order to enhance the separability, we used radial basis function (RBF) in Equation (1), which is also known as a Gaussian kernel, and has advantages over other kernel functions [72]. However, the γ parameter in RBF, besides the regularization parameter (*C*) of the SVM algorithm, requires grid optimization, which will determine the best *C*- γ pair for the best predictive performance.

(1) $$K\left(x,y\right)=\exp\left(\gamma ||x-y^{2}||\right)$$

We performed our grid search in the range of [10, 10^5^] and [10^−1^, 10^−6^], on an exponential scale, for C and γ, respectively. We utilized Python machine learning library, scikit-learn [73], to find the best parameters through 10-fold cross validation, in order to maximize the diagnostic accuracy, which is the ratio of correctly predicted samples, as described in Equation (2) below.

(2) $$accuracy=\frac{TP+TN}{TP+TN+FP+FN}$$

#### 4.4.5. Model Evaluation

In order to show the significance of the resulting accuracies, we did a permutation test over 1000 iterations, each of which used the best *C*- γ parameter pair under 10-fold cross validation. Finally, we calculated sensitivity, specificity, and AUC values for the ROC curves, for which we reported average values along with standard deviations for 10-fold cross validation.

### 4.5. Metabolites Pathways Enrichment Analysis

Metabolite set enrichment analysis (MSEA) was completed using MetaboAnalyst (v4.0, University of Alberta, AB, Canada) [74]. Metabolite names were converted to Human Metabolite Database (HMDB) identifiers. The raw data were subjected to quantile normalization, and cube root transformation and autoscaling. The pathway-associated metabolite set was the chosen metabolite library, and all compounds in this library were used. Pathways with a *p* value < 0.001 were considered to be significantly altered in the epileptic samples [75].

## 5. Conclusions

In conclusion, we report for the first time a targeted, quantitative metabolomic approach for profiling post-mortem human brain tissue from patients with ENOS. This study demonstrates the potential of metabolomics for identifying potential biomarkers of ENOS, while giving a previously unreported insight into the underlying pathogenesis of the disorder. Our study did encounter some limitations. Our sample size was modest, with 15 epileptic brain samples and 15 controls and future studies necessary to confirm our present preliminary data in more accessible biomatrices, such as blood. Furthermore, the extraction method used herein employed a lyophilization step, which may cause some of the more aromatic compounds to become depleted in the tissue extracts. While the effects are believed to be negligible (all samples were treated equally), this may account for minor differences in their relative concentrations. In addition, while we don’t completely understand the effect of PM interval on the brain’s metabolome, we must consider that there may be some degradation in the specimens which have longer PM delay times. As such, future studies are required to determine the effect of PM delay on degradation and the brain’s metabolome.

## Figures and Tables

**Table 1:** Results of the univariate analysis on important demographic factors.

	Controls	Epileptic	*p*-Value
*n*	15	15	
Age, mean (SD)	40.67(14.75)	40.8(15.06273)	0.98 ^a^
Gender			
Male	9	9	1 ^b^
Female	6	6	

^a^ One-way ANOVA ^b^ Chi-squared test.

## Data Availability

The metabolomics and metadata reported in this paper are available at MetaboLights Archive (https://www.ebi.ac.uk/metabolights/mysubmissions?status=PRIVATE) via the MetaboLights partner repository with the data set no. MTBLS1695. Username: ali.yilmaz@beaumont.org and study ID MTBLS1695.

## Supplementary Materials

The following are available online at https://www.mdpi.com/2218-1989/10/6/261/s1.
