# Supplementary material for: The Biochemical Profile of Post-Mortem Brain from People Who Suffered from Epilepsy Reveals Novel Insights into the Etiopathogenesis of the Disease
Source: Metabolites. 2020 Jun 23;10(6):261. doi: 10.3390/metabo10060261 (PMC7345034; doi:10.3390/metabo10060261)
Supplement: Supplementary file 1 [file metabolites-10-00261-s001.zip › supplementary/Supplementary Table 1.docx]

**Table 1.** Univariate Analysis Result for each metabolite concentration (μM) in epileptic brain and healthy controls, respectively.

| **Name** | **Mean (SD) of Controls** | **Mean (SD) of Epilepsy** | ***p*-Value** | ***q*-Value (FDR)** | **Fold Change** |
| --- | --- | --- | --- | --- | --- |
| **1-Methylhistidine** | **135.273 (46.022)** | **101.902 (39.890)** | **0.0428** | **0.04281** | **1.33** |
| 3-Hydroxyisobutyrate | 4.608 (4.108) | 3.728 (3.146) | 0.5156 | 0.51557 | 1.24 |
| **Acetic acid** | **239.005 (78.163)** | **313.873 (107.753)** | **0.0340 (W)** | **0.03402** | **-1.31** |
| **Ascorbic acid** | **454.468 (227.359)** | **293.129 (160.986)** | **0.033** | **0.03299** | **1.55** |
| **Acetoacetate** | **121.913 (32.349)** | **99.187 (13.074)** | **0.021** | **0.02102** | **1.23** |
| Creatine | 2733.916 (296.626) | 2692.682 (258.714) | 0.5182 (W) | 0.51824 | 1.02 |
| Dimethylamine | 115.611 (32.421) | 100.678 (13.584) | 0.1166 | 0.11655 | 1.15 |
| Choline | 124.016 (60.158) | 136.603 (58.374) | 0.5655 | 0.56551 | -1.1 |
| D-Glucose | 63.948 (51.491) | 69.062 (47.232) | 0.7087 (W) | 0.70871 | -1.08 |
| **Glycerol** | **506.863 (102.956)** | **578.591 (97.546)** | **0.0602** | **0.06017** | **-1.14** |
| Fumaric acid | 27.299 (9.363) | 32.435 (13.079) | 0.2265 | 0.2265 | -1.19 |
| Formate | 88.844 (41.654) | 101.315 (42.940) | 0.4065 (W) | 0.40647 | -1.14 |
| D-Galactose | 29.360 (21.870) | 30.199 (20.743) | 0.9149 | 0.91488 | -1.03 |
| Ethanolamine | 181.214 (61.722) | 209.142 (63.269) | 0.2705 (W) | 0.27047 | -1.15 |
| Hypoxanthine | 19.799 (9.833) | 18.358 (8.599) | 0.7392 (W) | 0.73918 | 1.08 |
| Tyrosine | 54.782 (26.100) | 56.042 (6.813) | 0.6475 (W) | 0.64751 | -1.02 |
| L-Phenylalanine | 84.294 (33.519) | 89.905 (19.249) | 0.2986 (W) | 0.29857 | -1.07 |
| L-Alanine | 424.946 (128.734) | 416.772 (74.523) | 0.6908 (W) | 0.69083 | 1.02 |
| L-Threonine | 236.174 (41.444) | 256.985 (65.799) | 0.3919 (W) | 0.39193 | -1.09 |
| Isoleucine | 108.853 (44.646) | 114.656 (14.388) | 0.1576 (W) | 0.15763 | -1.05 |
| L-Lysine | 128.755 (60.134) | 128.297 (27.763) | 0.5608 (W) | 0.56084 | 1 |
| **L-Serine** | **303.073 (50.629)** | **349.732 (59.474)** | **0.0282** | **0.02825** | **-1.15** |
| L-Leucine | 199.262 (84.670) | 216.410 (24.798) | 0.2191 (W) | 0.21909 | -1.09 |
| Methionine | 74.704 (33.745) | 82.016 (14.469) | 0.45 | 0.45005 | -1.1 |
| Valine | 174.234 (64.333) | 198.020 (28.309) | 0.2047 (W) | 0.20473 | -1.14 |
| Tryptophan | 19.984 (10.564) | 19.418 (4.455) | 0.5604 (W) | 0.5604 | 1.03 |
| **Citric Acid** | **44.052 (20.241)** | **31.143 (6.798)** | **0.0771 (W)** | **0.07707** | **1.41** |
| Glycine | 786.836 (198.286) | 787.582 (131.118) | 0.8804 (W) | 0.88041 | -1 |
| L-Glutamic acid | 4133.140 (349.598) | 4223.406 (0.000) | 0.3343 | 0.33428 | -1.02 |
| L-Glutamine | 2261.183 (298.516) | 2308.304 (427.490) | 0.8353 (W) | 0.8353 | -1.02 |
| Aspartate | 545.600 (102.981) | 572.729 (115.983) | 0.4346 (W) | 0.43459 | -1.05 |
| Creatinine | 134.493 (28.103) | 129.756 (15.968) | 0.8511 (W) | 0.85106 | 1.04 |
| L-Fucose | 11.934 (8.527) | 14.325 (5.991) | 0.3818 | 0.38179 | -1.2 |
| Myo-inositol | 1881.244 (240.301) | 1965.160 (266.353) | 0.3836 (W) | 0.38356 | -1.04 |
| Pyruvic acid | 80.083 (21.893) | 72.852 (16.053) | 0.1515 (W) | 0.15152 | 1.1 |
| Taurine | 311.728 (139.389) | 317.965 (86.762) | 0.5172 (W) | 0.51724 | -1.02 |
| Succinate | 221.206 (68.300) | 184.119 (55.755) | 0.1145 | 0.11448 | 1.2 |
| Pyroglutamic acid | 121.377 (42.800) | 139.145 (31.372) | 0.2053 | 0.20528 | -1.15 |
| Xanthine | 92.191 (39.552) | 88.915 (18.987) | 0.7754 | 0.77537 | 1.04 |
| Urea | 539.640 (1080.773) | 215.973 (232.008) | 0.4180 (W) | 0.418 | 2.5 |
| Uracil | 57.485 (22.863) | 55.118 (18.560) | 0.7579 | 0.75789 | 1.04 |
| 3-Hydroxybutyric acid | 67.907 (103.513) | 44.001 (24.787) | 0.7872 (W) | 0.78718 | 1.54 |
| 3-Methylhistidine | 42.059 (27.721) | 46.635 (28.665) | 0.6602 | 0.66018 | -1.11 |
| Malonate | 35.767 (32.364) | 26.826 (8.977) | 0.7872 (W) | 0.78723 | 1.33 |
| **3-Hydroxyisovaleric acid** | **3.872 (10.540)** | **3.070 (2.129)** | **0.0340 (W)** | **0.03398** | **1.26** |
| Isopropyl alcohol | 5.272 (2.203) | 5.331 (1.839) | 0.9381 | 0.93806 | -1.01 |
| Acetone | 4.683 (2.316) | 4.034 (1.545) | 0.3741 | 0.37409 | 1.16 |
| Isobutyric acid | 3.976 (2.643) | 4.910 (2.510) | 0.3294 | 0.32942 | -1.23 |
| Methanol | 389.010 (260.772) | 511.647 (669.419) | 0.8679 (W) | 0.86789 | -1.32 |
| Propylene glycol | 2.978 (2.676) | 2.335 (1.712) | 0.4804 (W) | 0.48039 | 1.28 |
| Dimethyl sulfone | 10.876 (3.499) | 10.745 (4.600) | 0.7239 (W) | 0.72388 | 1.01 |
| 2-Hydroxyisovalerate | 19.677 (31.764) | 13.600 (5.621) | 0.2895 (W) | 0.2895 | 1.45 |
| 3-Hydroxyisobutyrate.1 | 11.088 (5.644) | 10.386 (3.763) | 0.9171 (W) | 0.91709 | 1.07 |
| 3-Hydroxyisovalerate | 8.263 (11.938) | 6.275 (2.284) | 0.3609 (W) | 0.36091 | 1.32 |
| 4-Aminobutyrate | 963.786 (236.481) | 866.107 (139.267) | 0.1054 (W) | 0.10545 | 1.11 |
| Adenine | 501.935 (266.278) | 396.135 (256.090) | 0.3822 (W) | 0.38225 | 1.27 |
| ADP | 11.813 (5.374) | 9.457 (4.849) | 0.1246 (W) | 0.12461 | 1.25 |
| **AMP** | **36.570 (22.343)** | **16.630 (9.331)** | **0.0026 (W)** | **0.00261** | **2.2** |
| ATP | 10.611 (15.572) | 7.853 (4.968) | 0.9503 (W) | 0.95033 | 1.35 |
| Fucose | 18.194 (7.628) | 16.699 (7.486) | 0.4799 (W) | 0.4799 | 1.09 |
| GTP | 5.514 (3.313) | 4.745 (2.009) | 0.6780 (W) | 0.67796 | 1.16 |
| Histamine | 129.003 (58.077) | 115.944 (44.977) | 0.4968 | 0.49679 | 1.11 |
| IMP | 34.677 (53.702) | 30.709 (55.364) | 0.1102 (W) | 0.11021 | 1.13 |
| Inosine | 153.009 (77.252) | 119.133 (66.906) | 0.2710 (W) | 0.271 | 1.28 |
| N-Acetylaspartate | 1222.108 (614.999) | 1391.922 (310.498) | 0.8584 (W) | 0.85843 | -1.14 |
| **Niacinamide** | **114.992 (38.403)** | **91.671 (18.062)** | **0.046** | **0.04599** | **1.25** |
| O-Acetylcarnitine | 5.344 (1.895) | 5.269 (1.803) | 0.9114 | 0.91136 | 1.01 |
| O-Acetylcholine | 140.869 (65.508) | 174.037 (58.819) | 0.3187 (W) | 0.3187 | -1.24 |
| **O-Phosphocholine** | **69.079 (51.496)** | **33.009 (9.717)** | **0.0047 (W)** | **0.072** | **2.09** |
| Pantothenate | 63.618 (26.639) | 63.938 (13.626) | 0.4528 (W) | 0.45283 | -1.01 |
| sn-Glycero-3-phosphocholine | 246.407 (94.299) | 199.364 (79.963) | 0.0926 (W) | 0.09258 | 1.24 |
| C0 | 16.337 (3.034) | 17.706 (3.701) | 0.277 | 0.27704 | -1.08 |
| C2 | 1.954 (1.096) | 1.729 (0.586) | 0.9503 (W) | 0.95031 | 1.13 |
| C3 | 0.086 (0.052) | 0.057 (0.026) | 0.1043 (W) | 0.10426 | 1.51 |
| C3-DC C4-OH | 0.077 (0.045) | 0.067 (0.023) | 0.7072 (W) | 0.70724 | 1.15 |
| **C4** | **0.261 (0.195)** | **0.144 (0.099)** | **0.0259 (W)** | **0.02588** | **1.82** |
| **C5** | **0.084 (0.067)** | **0.046 (0.017)** | **0.0434 (W)** | **0.04338** | **1.82** |
| C5-DC C6-OH | 0.020 (0.010) | 0.014 (0.007) | 0.1052 (W) | 0.10524 | 1.41 |
| **C5-OH C3-DC-M** | **0.124 (0.057)** | **0.086 (0.047)** | **0.0286 (W)** | **0.02857** | **1.44** |
| **C6 C41-DC** | **0.037 (0.011)** | **0.025 (0.015)** | **0.0085 (W)** | **0.00854** | **1.48** |
| C14 | 0.077 (0.051) | 0.063 (0.016) | 1.0000 (W) | 1 | 1.22 |
| C141 | 0.011 (0.007) | 0.007 (0.004) | 0.1049 (W) | 0.10486 | 1.5 |
| C16 | 0.125 (0.075) | 0.111 (0.044) | 0.8840 (W) | 0.88397 | 1.12 |
| C161 | 0.025 (0.013) | 0.020 (0.008) | 0.1540 (W) | 0.15397 | 1.26 |
| C18 | 0.076 (0.046) | 0.058 (0.020) | 0.2432 (W) | 0.24322 | 1.31 |
| C181 | 0.086 (0.053) | 0.087 (0.063) | 0.6618 (W) | 0.66181 | -1.01 |
| Arg | 73.738 (36.260) | 76.716 (10.678) | 0.1635 (W) | 0.1635 | -1.04 |
| **Asn** | **29.877 (17.328)** | **34.159 (6.959)** | **0.0483 (W)** | **0.04834** | **-1.14** |
| His | 55.040 (25.675) | 59.084 (10.777) | 0.5804 | 0.58042 | -1.07 |
| Orn | 8.978 (4.924) | 10.313 (5.636) | 0.4954 | 0.49538 | -1.15 |
| Pro | 145.484 (72.387) | 168.729 (49.797) | 0.1349 (W) | 0.13495 | -1.16 |
| Ac-Orn | 0.147 (0.183) | 0.178 (0.135) | 0.2449 (W) | 0.24491 | -1.21 |
| ADMA | 0.245 (0.299) | 0.177 (0.082) | 0.7081 (W) | 0.70809 | 1.38 |
| alpha-AAA | 1.313 (0.544) | 1.355 (0.465) | 0.8217 | 0.8217 | -1.03 |
| Carnosine | 0.797 (0.624) | 0.640 (0.283) | 0.6333 (W) | 0.63325 | 1.25 |
| DOPA | 0.073 (0.029) | 0.080 (0.029) | 0.5291 | 0.52912 | -1.09 |
| Histamine.1 | 0.278 (0.227) | 0.224 (0.093) | 0.5185 (W) | 0.51846 | 1.24 |
| Kynurenine | 0.233 (0.195) | 0.209 (0.093) | 0.7860 (W) | 0.78602 | 1.11 |
| Met-SO | 2.018 (1.094) | 2.340 (0.701) | 0.2535 (W) | 0.25349 | -1.16 |
| Nitro-Tyr | 0.227 (0.086) | 0.201 (0.061) | 0.6762 (W) | 0.67622 | 1.13 |
| PEA | 0.057 (0.025) | 0.049 (0.020) | 0.3553 | 0.35533 | 1.16 |
| Putrescine | 10.303 (5.085) | 10.330 (7.037) | 0.6328 (W) | 0.63276 | -1 |
| Sarcosine | 1.314 (0.547) | 1.275 (0.637) | 0.5610 (W) | 0.56101 | 1.03 |
| SDMA | 0.431 (0.278) | 0.407 (0.135) | 0.5599 (W) | 0.55992 | 1.06 |
| Serotonin | 0.039 (0.016) | 0.041 (0.034) | 0.7673 | 0.76733 | -1.07 |
| Spermidine | 28.212 (7.755) | 31.565 (11.898) | 0.6614 (W) | 0.66145 | -1.12 |
| Spermine | 3.980 (2.252) | 5.004 (4.288) | 0.7399 (W) | 0.73985 | -1.26 |
| t4-OH-Pro | 4.352 (1.527) | 4.145 (1.177) | 0.9503 (W) | 0.95027 | 1.05 |
| Taurine. | 265.799 (138.878) | 330.158 (133.730) | 0.2066 | 0.20662 | -1.24 |
| lysoPC a C16:0 | 38.749 (12.306) | 40.159 (5.251) | 0.1832 (W) | 0.18319 | -1.04 |
| lysoPC a C16:1 | 3.924 (0.981) | 4.250 (0.861) | 0.3416 | 0.34162 | -1.08 |
| lysoPC a C17:0 | 0.570 (0.113) | 0.608 (0.137) | 0.4146 | 0.41461 | -1.07 |
| lysoPC a C18:0 | 8.952 (2.697) | 9.193 (1.424) | 0.7618 | 0.76183 | -1.03 |
| lysoPC a C18:1 | 36.299 (11.794) | 36.610 (5.857) | 0.2521 (W) | 0.25206 | -1.01 |
| lysoPC a C18:2 | 2.091 (0.655) | 1.944 (0.377) | 0.4596 | 0.45964 | 1.08 |
| lysoPC a C20:3 | 1.460 (0.512) | 1.430 (0.336) | 0.5453 (W) | 0.54531 | 1.02 |
| lysoPC a C20:4 | 8.998 (2.385) | 9.246 (2.617) | 0.7879 | 0.78794 | -1.03 |
| lysoPC a C24:0 | 0.697 (0.158) | 0.696 (0.122) | 0.9888 | 0.98884 | 1 |
| lysoPC a C26:0 | 0.924 (0.169) | 0.913 (0.142) | 0.8468 | 0.84681 | 1.01 |
| lysoPC a C26:1 | 0.423 (0.166) | 0.393 (0.114) | 0.9834 (W) | 0.98341 | 1.07 |
| lysoPC a C28:0 | 0.751 (0.192) | 0.718 (0.112) | 0.5694 | 0.56941 | 1.05 |
| lysoPC a C28:1 | 0.726 (0.080) | 0.695 (0.084) | 0.3118 | 0.31184 | 1.04 |
| PC aa C24:0 | 0.469 (0.122) | 0.465 (0.074) | 0.9128 | 0.91284 | 1.01 |
| PC aa C26:0 | 0.905 (0.137) | 0.897 (0.088) | 0.8468 | 0.8468 | 1.01 |
| PC aa C28:1 | 0.734 (0.063) | 0.705 (0.065) | 0.2256 | 0.22557 | 1.04 |
| PC aa C30:0 | 48.880 (20.638) | 42.453 (4.803) | 0.6926 (W) | 0.69257 | 1.15 |
| PC aa C32:0 | 208.149 (66.217) | 219.978 (32.748) | 0.1513 (W) | 0.15134 | -1.06 |
| PC aa C32:1 | 130.749 (31.302) | 134.883 (26.144) | 0.6976 | 0.69763 | -1.03 |
| PC aa C32:2 | 5.003 (1.167) | 5.097 (1.012) | 0.5886 (W) | 0.58858 | -1.02 |
| PC aa C32:3 | 0.503 (0.074) | 0.470 (0.095) | 0.298 | 0.29796 | 1.07 |
| PC aa C34:1 | 179.380 (49.333) | 184.021 (25.798) | 0.4541 (W) | 0.45414 | -1.03 |
| PC aa C34:2 | 93.403 (30.687) | 84.573 (13.853) | 0.9008 (W) | 0.90075 | 1.1 |
| PC aa C34:3 | 4.093 (0.873) | 3.671 (0.697) | 0.1544 | 0.15438 | 1.11 |
| PC aa C34:4 | 1.167 (0.225) | 1.098 (0.188) | 0.3701 | 0.37008 | 1.06 |
| PC aa C36:0 | 21.542 (5.853) | 21.936 (4.022) | 0.8316 | 0.83158 | -1.02 |
| PC aa C36:1 | 194.275 (53.356) | 205.619 (30.640) | 0.1886 (W) | 0.18864 | -1.06 |
| PC aa C36:2 | 137.539 (35.341) | 139.648 (25.033) | 0.8518 | 0.8518 | -1.02 |
| PC aa C36:3 | 42.271 (13.659) | 37.060 (7.119) | 0.4166 (W) | 0.41659 | 1.14 |
| PC aa C36:4 | 138.275 (43.926) | 136.567 (29.665) | 0.9016 | 0.90159 | 1.01 |
| PC aa C36:5 | 3.802 (1.126) | 3.793 (1.609) | 0.7708 (W) | 0.77078 | 1 |
| PC aa C36:6 | 0.637 (0.112) | 0.623 (0.144) | 0.6172 (W) | 0.61718 | 1.02 |
| PC aa C38:0 | 2.667 (0.267) | 2.532 (0.223) | 0.1429 | 0.14294 | 1.05 |
| PC aa C38:3 | 20.900 (6.781) | 20.847 (3.427) | 0.5728 (W) | 0.57281 | 1 |
| PC aa C38:4 | 142.557 (49.339) | 142.993 (39.928) | 0.8514 (W) | 0.85144 | -1 |
| PC aa C38:5 | 41.573 (13.693) | 39.494 (7.859) | 0.9172 (W) | 0.91715 | 1.05 |
| PC aa C38:6 | 77.843 (27.856) | 70.637 (16.561) | 0.3965 | 0.39647 | 1.1 |
| PC aa C40:1 | 0.728 (0.046) | 0.721 (0.051) | 0.7007 | 0.70067 | 1.01 |
| PC aa C40:2 | 2.070 (0.404) | 2.016 (0.385) | 0.7079 | 0.70794 | 1.03 |
| PC aa C40:3 | 2.157 (0.413) | 2.037 (0.288) | 0.362 | 0.36202 | 1.06 |
| PC aa C40:4 | 21.422 (6.840) | 19.710 (4.346) | 0.9170 (W) | 0.91703 | 1.09 |
| PC aa C40:5 | 10.646 (2.988) | 10.036 (1.698) | 0.8187 (W) | 0.81867 | 1.06 |
| PC aa C40:6 | 47.748 (12.548) | 45.177 (6.752) | 0.4922 | 0.49216 | 1.06 |
| PC aa C42:0 | 0.362 (0.111) | 0.346 (0.072) | 0.6537 | 0.65366 | 1.04 |
| PC aa C42:1 | 0.719 (0.140) | 0.692 (0.110) | 0.5714 | 0.57136 | 1.04 |
| PC aa C42:2 | 2.253 (0.935) | 2.169 (0.468) | 0.6933 (W) | 0.69326 | 1.04 |
| PC aa C42:4 | 1.106 (0.256) | 1.105 (0.203) | 0.4157 (W) | 0.4157 | 1 |
| PC aa C42:5 | 0.905 (0.130) | 0.868 (0.118) | 0.4222 | 0.42223 | 1.04 |
| PC aa C42:6 | 0.550 (0.112) | 0.507 (0.077) | 0.2405 | 0.24048 | 1.08 |
| PC ae C30:0 | 0.994 (0.159) | 0.973 (0.145) | 0.7038 | 0.70378 | 1.02 |
| PC ae C30:1 | 0.274 (0.062) | 0.260 (0.042) | 0.4645 | 0.46453 | 1.06 |
| PC ae C30:2 | 0.186 (0.056) | 0.161 (0.036) | 0.1596 | 0.15963 | 1.15 |
| PC ae C32:1 | 7.749 (1.416) | 7.656 (1.203) | 0.8474 | 0.84739 | 1.01 |
| PC ae C32:2 | 0.871 (0.174) | 0.862 (0.143) | 0.8837 | 0.88375 | 1.01 |
| PC ae C34:0 | 11.632 (4.503) | 11.502 (4.061) | 0.9834 (W) | 0.98338 | 1.01 |
| PC ae C34:1 | 64.082 (15.856) | 64.480 (15.279) | 0.7870 (W) | 0.787 | -1.01 |
| PC ae C34:2 | 12.580 (3.780) | 13.161 (3.634) | 0.671 | 0.67105 | -1.05 |
| PC ae C34:3 | 0.923 (0.209) | 0.835 (0.125) | 0.1724 | 0.17243 | 1.11 |
| PC ae C36:0 | 4.789 (2.782) | 5.081 (1.853) | 0.3090 (W) | 0.30899 | -1.06 |
| PC ae C36:1 | 90.843 (29.528) | 93.936 (23.468) | 0.6626 (W) | 0.66262 | -1.03 |
| PC ae C36:2 | 13.558 (4.234) | 14.079 (3.247) | 0.7081 | 0.70808 | -1.04 |
| PC ae C36:3 | 7.581 (3.377) | 7.027 (2.265) | 0.6017 | 0.60169 | 1.08 |
| PC ae C36:4 | 4.435 (0.926) | 4.491 (0.879) | 0.8666 | 0.8666 | -1.01 |
| **PC ae C36:5** | **2.421 (0.623)** | **2.042 (0.330)** | **0.01005 (W)** | **0.10054** | **1.19** |
| PC ae C38:0 | 1.957 (0.412) | 1.928 (0.269) | 0.8217 | 0.82166 | 1.01 |
| PC ae C38:1 | 11.975 (5.312) | 12.560 (2.833) | 0.1632 (W) | 0.16322 | -1.05 |
| PC ae C38:2 | 6.287 (1.825) | 6.074 (1.077) | 0.699 | 0.69898 | 1.04 |
| PC ae C38:3 | 4.575 (0.962) | 4.494 (0.753) | 1.0000 (W) | 1 | 1.02 |
| PC ae C38:4 | 6.568 (1.447) | 6.425 (1.467) | 0.7901 | 0.79011 | 1.02 |
| PC ae C38:5 | 4.645 (0.940) | 4.645 (0.683) | 0.5887 (W) | 0.5887 | -1 |
| PC ae C38:6 | 2.539 (0.536) | 2.445 (0.468) | 0.6152 | 0.61522 | 1.04 |
| PC ae C40:1 | 3.075 (0.595) | 2.976 (0.563) | 0.7870 (W) | 0.78702 | 1.03 |
| PC ae C40:2 | 6.439 (1.626) | 7.152 (1.506) | 0.2232 | 0.22322 | -1.11 |
| PC ae C40:3 | 2.876 (0.570) | 2.964 (0.472) | 0.6497 | 0.64975 | -1.03 |
| PC ae C40:4 | 2.978 (0.616) | 2.893 (0.541) | 0.6925 | 0.69248 | 1.03 |
| PC ae C40:5 | 3.305 (0.552) | 3.155 (0.409) | 0.4045 | 0.40454 | 1.05 |
| PC ae C40:6 | 3.143 (0.333) | 3.191 (0.379) | 0.7139 | 0.71386 | -1.02 |
| **PC ae C42:0** | **1.740 (0.246)** | **1.612 (0.150)** | **0.0496** | **0.04672** | **1.08** |
| PC ae C42:1 | 1.735 (0.204) | 1.626 (0.242) | 0.1628 (W) | 0.16284 | 1.07 |
| PC ae C42:2 | 1.725 (0.213) | 1.658 (0.282) | 0.3695 (W) | 0.36951 | 1.04 |
| PC ae C42:3 | 2.798 (0.526) | 2.625 (0.429) | 0.8352 (W) | 0.83518 | 1.07 |
| PC ae C42:4 | 0.486 (0.102) | 0.482 (0.064) | 0.8838 | 0.88382 | 1.01 |
| PC ae C42:5 | 1.058 (0.123) | 1.009 (0.113) | 0.2663 | 0.26633 | 1.05 |
| PC ae C44:3 | 0.593 (0.065) | 0.592 (0.087) | 0.6602 (W) | 0.66019 | 1 |
| PC ae C44:4 | 0.450 (0.076) | 0.421 (0.099) | 0.3744 | 0.37437 | 1.07 |
| PC ae C44:5 | 1.095 (0.282) | 1.044 (0.199) | 0.5728 | 0.57275 | 1.05 |
| PC ae C44:6 | 0.166 (0.041) | 0.151 (0.021) | 0.1467 (W) | 0.14669 | 1.1 |
| SM OH C14:1 | 0.618 (0.121) | 0.563 (0.134) | 0.2489 | 0.24886 | 1.1 |
| SM OH C16:1 | 3.815 (0.941) | 3.551 (0.741) | 0.4531 (W) | 0.45308 | 1.07 |
| SM OH C22:1 | 1.508 (0.393) | 1.369 (0.399) | 0.3422 | 0.34216 | 1.1 |
| SM OH C22:2 | 4.363 (1.740) | 4.856 (1.154) | 0.3675 | 0.36748 | -1.11 |
| SM OH C24:1 | 0.334 (0.278) | 0.342 (0.219) | 0.936 | 0.936 | -1.02 |
| SM C16:0 | 40.315 (15.246) | 35.465 (4.361) | 0.4407 (W) | 0.44073 | 1.14 |
| SM C16:1 | 1.928 (0.415) | 1.824 (0.200) | 0.3886 | 0.38858 | 1.06 |
| SM C18:0 | 241.012 (110.630) | 235.306 (52.318) | 0.6928 (W) | 0.6928 | 1.02 |
| SM C18:1 | 75.319 (28.616) | 69.621 (15.613) | 0.8512 (W) | 0.85121 | 1.08 |
| SM C20:2 | 0.117 (0.054) | 0.111 (0.050) | 0.7649 | 0.76485 | 1.05 |
| SM C24:0 | 12.695 (3.197) | 12.209 (2.220) | 0.9333 (W) | 0.93328 | 1.04 |
| SM C24:1 | 101.579 (35.262) | 111.717 (34.532) | 0.433 | 0.43298 | -1.1 |
| SM C26:0 | 0.430 (0.087) | 0.461 (0.146) | 0.4861 | 0.48607 | -1.07 |
| SM C26:1 | 5.609 (1.105) | 5.842 (1.374) | 0.6126 | 0.61265 | -1.04 |
| H1 | 1153.710 (519.538) | 1213.829 (255.158) | 0.1239 (W) | 0.12389 | -1.05 |

* *p*-value is calculated with t-test as a default, ** *p*-value with (W) is calculated by the Wilcoxon Mann Whitney test.
