# Supplementary material for: The Biochemical Profile of Post-Mortem Brain from People Who Suffered from Epilepsy Reveals Novel Insights into the Etiopathogenesis of the Disease
Source: Metabolites. 2020 Jun 23;10(6):261. doi: 10.3390/metabo10060261 (PMC7345034; doi:10.3390/metabo10060261)
Supplement: Supplementary file 1 [file metabolites-10-00261-s001.zip › supplementary/Supplementary Table 2.docx]

**Supplementary Table 2.** Top-five metabolite panels suggested by CFS and LASSO and corresponding performance metrics for the support vector machine (SVM) and logistic regression (LR) models using all metabolites, common metabolites selected by both CSF and LASSO, respectively.

| **PANEL OF METABOLITES** | | | | |
| --- | --- | --- | --- | --- |
| **CFS based metabolites** | | | **LASSO based metabolites** | |
| Glycerol | | | AMP | |
| L-Fucose | | | O-Acetylcholine | |
| Isobutyric.acid | | | L-Fucose | |
| AMP | | | Isobutyric acid | |
| O-Acetylcholine | | | Glycerol | |
| PC ae C 40:2 | | | 1-Methylhistidine | |
| **MODEL PERFORMANCE** | | | | |
| **Models** | **Accuracy** | **AUC** | **Sensitivity** | **Specificity** |
| **All metabolites (SVM)** | 0.78 | 0.77 | 0.67 | 0.65 |
| **CFS-LASSO (SVM)** | 0.83 | 0.90 | 0.85 | 0.89 |
| **All metabolites (LR)** | 0.75 | 0.68 | 0.66 | 0.64 |
| **CFS-LASSO (LR)** | 0.80 | 0.77 | 0.81 | 0.73 |
