# Supplementary material for: The Biochemical Profile of Post-Mortem Brain from People Who Suffered from Epilepsy Reveals Novel Insights into the Etiopathogenesis of the Disease
Source: Metabolites. 2020 Jun 23;10(6):261. doi: 10.3390/metabo10060261 (PMC7345034; doi:10.3390/metabo10060261)
Supplement: Supplementary file 1 [file metabolites-10-00261-s001.zip › supplementary/Supplementary Table 3.docx]

**Table S3.** The results from pathway topology analysis showing significantly perturbed pathways when healthy controls are compared to ENOS samples.

| **Pathway Name** | **Total** | **Hits** | **P value** | **FDR** |
| --- | --- | --- | --- | --- |
| Ethanol Degradation | 19 | 3 | 0.003721 | 0.1644 |
| Butyrate Metabolism | 19 | 4 | 0.00523 | 0.1644 |
| Mitochondrial Beta-Oxidation of Medium Chain Saturated Fatty Acids | 27 | 2 | 0.0077 | 0.1644 |
| Selenoamino Acid Metabolism | 28 | 5 | 0.011779 | 0.1644 |
| Mitochondrial Beta-Oxidation of Long Chain Saturated Fatty Acids | 28 | 4 | 0.014334 | 0.1644 |
| Mitochondrial Beta-Oxidation of Short Chain Saturated Fatty Acids | 27 | 3 | 0.015447 | 0.1644 |
| Pentose Phosphate Pathway | 29 | 3 | 0.019607 | 0.1644 |
| Riboflavin Metabolism | 20 | 3 | 0.019607 | 0.1644 |
| Thiamine Metabolism | 9 | 3 | 0.019607 | 0.1644 |
| Propanoate Metabolism | 42 | 5 | 0.022782 | 0.1644 |
| Homocysteine Degradation | 9 | 1 | 0.025123 | 0.1644 |
| Phenylacetate Metabolism | 9 | 3 | 0.025697 | 0.1644 |
| Phenylalanine and Tyrosine Metabolism | 28 | 7 | 0.02916 | 0.1644 |
| Nicotinate and Nicotinamide Metabolism | 37 | 6 | 0.029481 | 0.1644 |
| Fatty acid Metabolism | 43 | 4 | 0.032259 | 0.1644 |
| Fatty Acid Biosynthesis | 35 | 4 | 0.033028 | 0.1644 |
| Pantothenate and CoA Biosynthesis | 21 | 4 | 0.033271 | 0.1644 |
| Pyruvate Metabolism | 48 | 7 | 0.037274 | 0.1739 |
| Cysteine Metabolism | 26 | 5 | 0.045751 | 0.2022 |
