# Supplementary material for: The Biochemical Profile of Post-Mortem Brain from People Who Suffered from Epilepsy Reveals Novel Insights into the Etiopathogenesis of the Disease
Source: Metabolites. 2020 Jun 23;10(6):261. doi: 10.3390/metabo10060261 (PMC7345034; doi:10.3390/metabo10060261)
Supplement: Supplementary file 1 [file metabolites-10-00261-s001.zip › supplementary/Supplementary Table 4.docx]

**Supplementary Table 4:** Clinical and demographic information for each patient and corresponding controls.

| **UMBN** | **IC** | **Sec #** | **HBSAG** | **HIV** | **DISORDER** | **CDEATHOFF** | **AGE** | **Sex** | **Race** | **PM Delay** |
| --- | --- | --- | --- | --- | --- | --- | --- | --- | --- | --- |
| 1310 | Y | 3 | None | None | ENOS | Drowning due to Epilepsy | 36 | F | White | 35 |
| 5607 | Y | 9 | Negative | Negative | ENOS | Combined Drug Intoxication | 19 | M | White | 29 |
| 5612 | Y | 9 | Negative | Negative | ENOS | Atherosclerosis Cardiovascular Disease | 52 | M | White | 20 |
| 5664 | Y | 9 |  |  | ENOS | Atherosclerotic Cardiovascular Disease | 51 | M | Black or African-American | 27 |
| 5695 | Y | 7 | Negative | Negative | ENOS | Hepatic Steatosis | 50 | M | White | 30 |
| 5763 | Y | 7 | None | None | ENOS | Seizure Disorder, complicated positional asphyxia | 32 | F | White | 28 |
| 5812 | Y | 7 | None | None | ENOS | Drowning | 49 | M | White | 17 |
| 5839 | Y | 9 | None | None | ENOS | Epilepsy complicated by drowning | 21 | F | White | 18 |
| 5850 | Y | 7 | None | None | ENOS | Sudden Unexpected Death in Epilepsy | 27 | F | Black or African-American | 30 |
| 5876 | Y | 7 | None | None | ENOS | Atherosclerotic Cardiovascular Disease | 49 | M | White | 26 |
| 5884 | Y | 7 | Negative | Negative | ENOS | Seizure | 50 | F | Asian | 19 |
| 5892 | Y | 10 | Negative | Negative | ENOS | Seizure Disorder | 33 | Male | Black or African-American | 25 |
| 5894 | Y | 7 | None | None | ENOS | Atherosclerotic Cardiovascular Disease | 75 | F | White | 21 |
| 5916 | Y | 7 | Negative | Negative | ENOS | Hypertensive Cardiovascular Disease | 43 | M | White | 22 |
| 5929 | Y | 7 | Negative | Negative | ENOS | Positional asphyxia associated with seizure | 25 | M | Black or African-American | 25 |
| 605 | Y | 3 | None | Negative | Control | Asthma | 25 | M | Black or African-American | 19 |
| 1579 | Y | 7 | Negative | Negative | Control | Asthma | 21 | F | Black or African-American | 22 |
| 1595 | Y | 7 | Negative | Negative | Control | Chronic Alcoholism | 43 | M | Black or African-American | 18 |
| 5236 | Y | 9 | Negative | Negative | Control | Cardiac Arrhythmia due to Cardiomegaly | 27 | F | Black or African-American | 11 |
| 5237 | Y | 7 | Negative | Negative | Control | HASCVD | 52 | M | White | 13 |
| 5250 | Y | 3 | None | Negative | Control | HCVD; myxoid heart valves; obesity | 51 | M | White | 25 |
| 5604 | Y | 7 |  |  | Control | Multiple Injuries | 75 | F | White | 20 |
| 5706 | Y | 9 | None | None | Control | Atherosclerotic Cardiovascular Disease | 50 | F | White | 25 |
| 5849 | Y | 9 | None | None | Control | Atherosclerotic Cardiovascular Disease | 36 | F | White | 23 |
| 5893 | Y | 7 | Negative | Negative | Control | Dilated Cardiomegaly | 19 | M | White | 11 |
| 6059 | Y | 7 | None | None | Control | Fentanyl Intoxication and ASCVD | 33 | M | Black or African-American | 24 |
| 6060 | Y | 9 | None | None | Control | Cardiac tamponade | 49 | M | Black or African-American | 22 |
| 6062 | Y | 9 | Negative | Negative | Control | Mixed Drug Intoxication | 32 | F | White | 8 |
| 6139 | Y | 9 | Negative | Negative | Control | Acute Myocardial Infarct;Acute Coronary Artery | 50 | M | White | 22 |
| 6173 | Y | 7 | None | None | Control | Atherosclerotic Cardiovascular Disease | 49 | M |  | 13 |
