# Supplementary figures and images for: The Biochemical Profile of Post-Mortem Brain from People Who Suffered from Epilepsy Reveals Novel Insights into the Etiopathogenesis of the Disease
Source: Metabolites. 2020 Jun 23;10(6):261. doi: 10.3390/metabo10060261 (PMC7345034; doi:10.3390/metabo10060261)

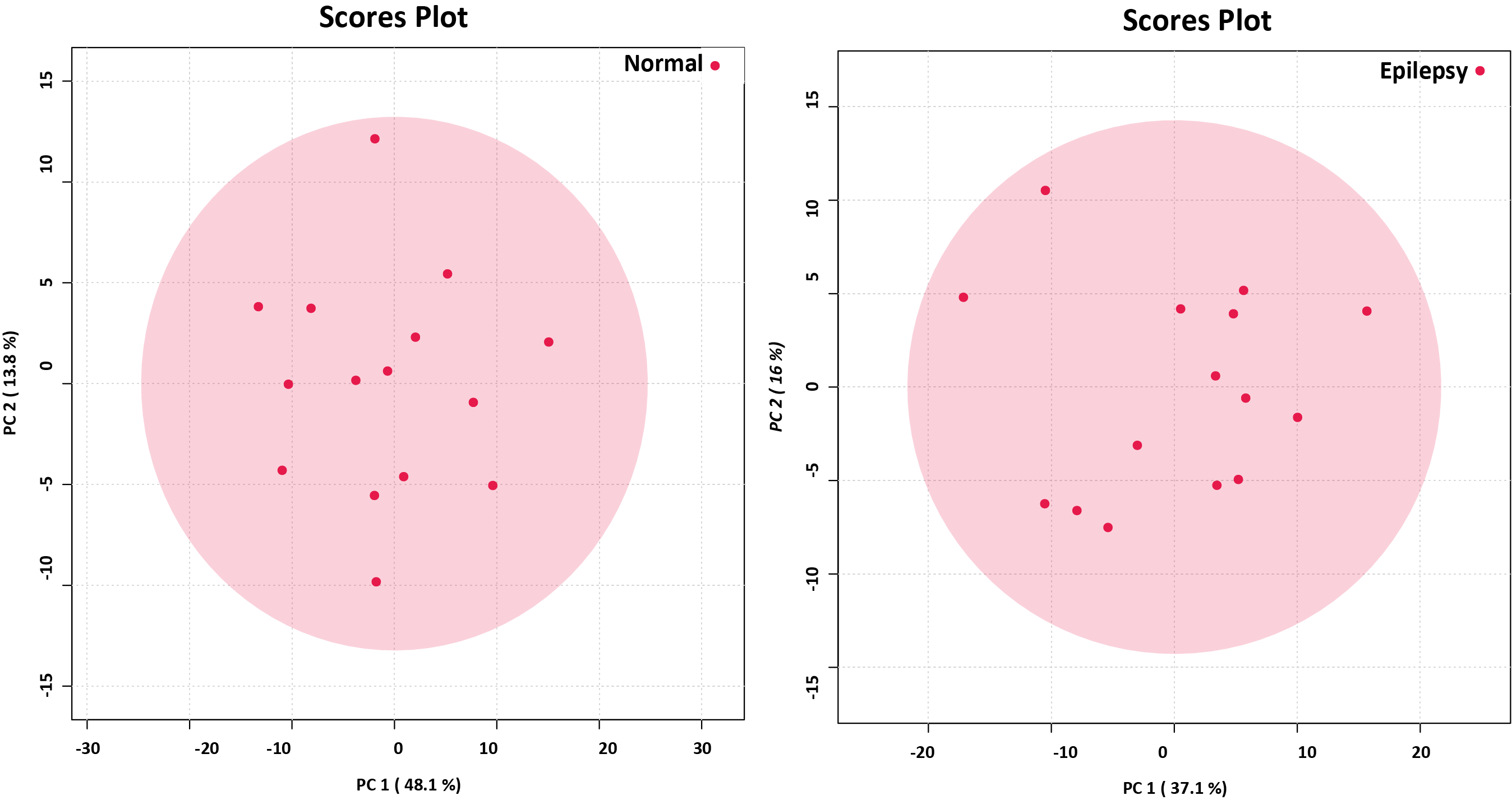

Supplement: Supplementary file 1 [file metabolites-10-00261-s001.zip › supplementary/Supplementary Figure 1.png]

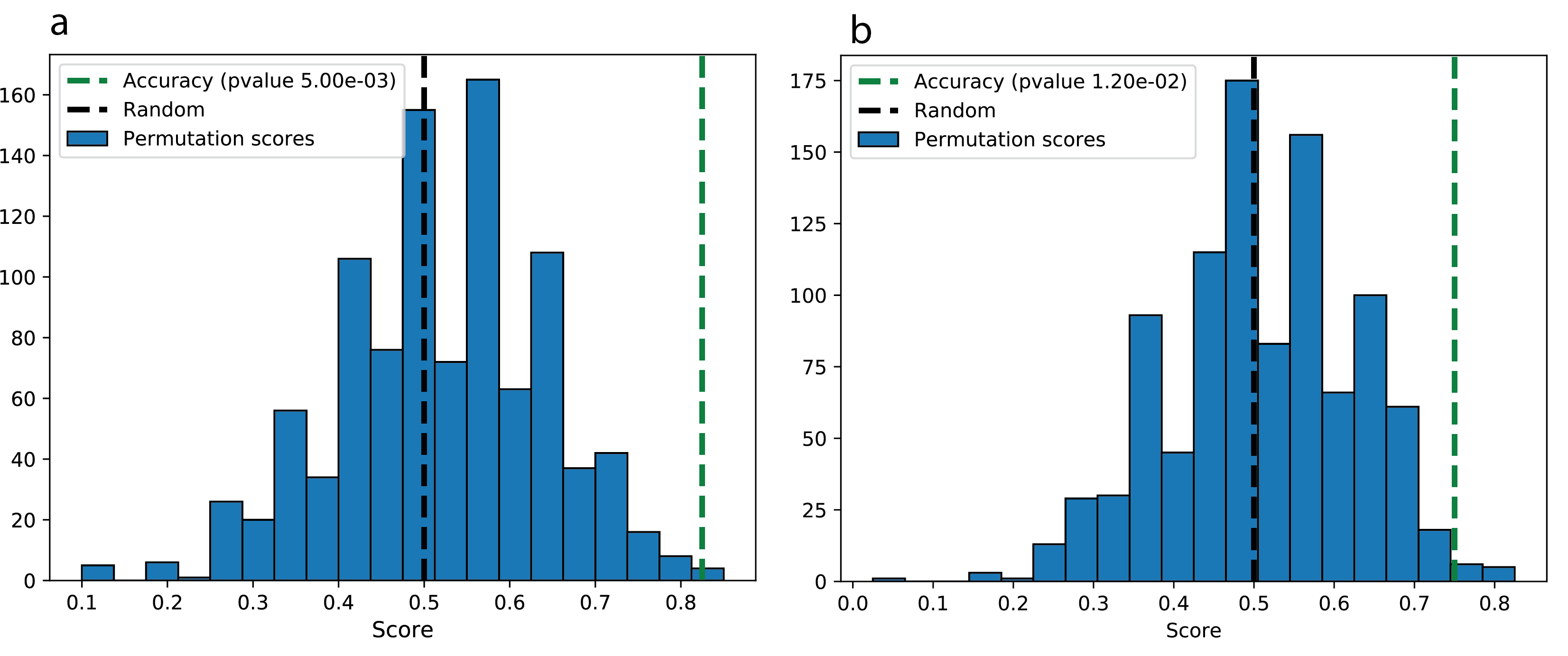

Supplement: Supplementary file 1 [file metabolites-10-00261-s001.zip › supplementary/Supplementary Figure 2.png]

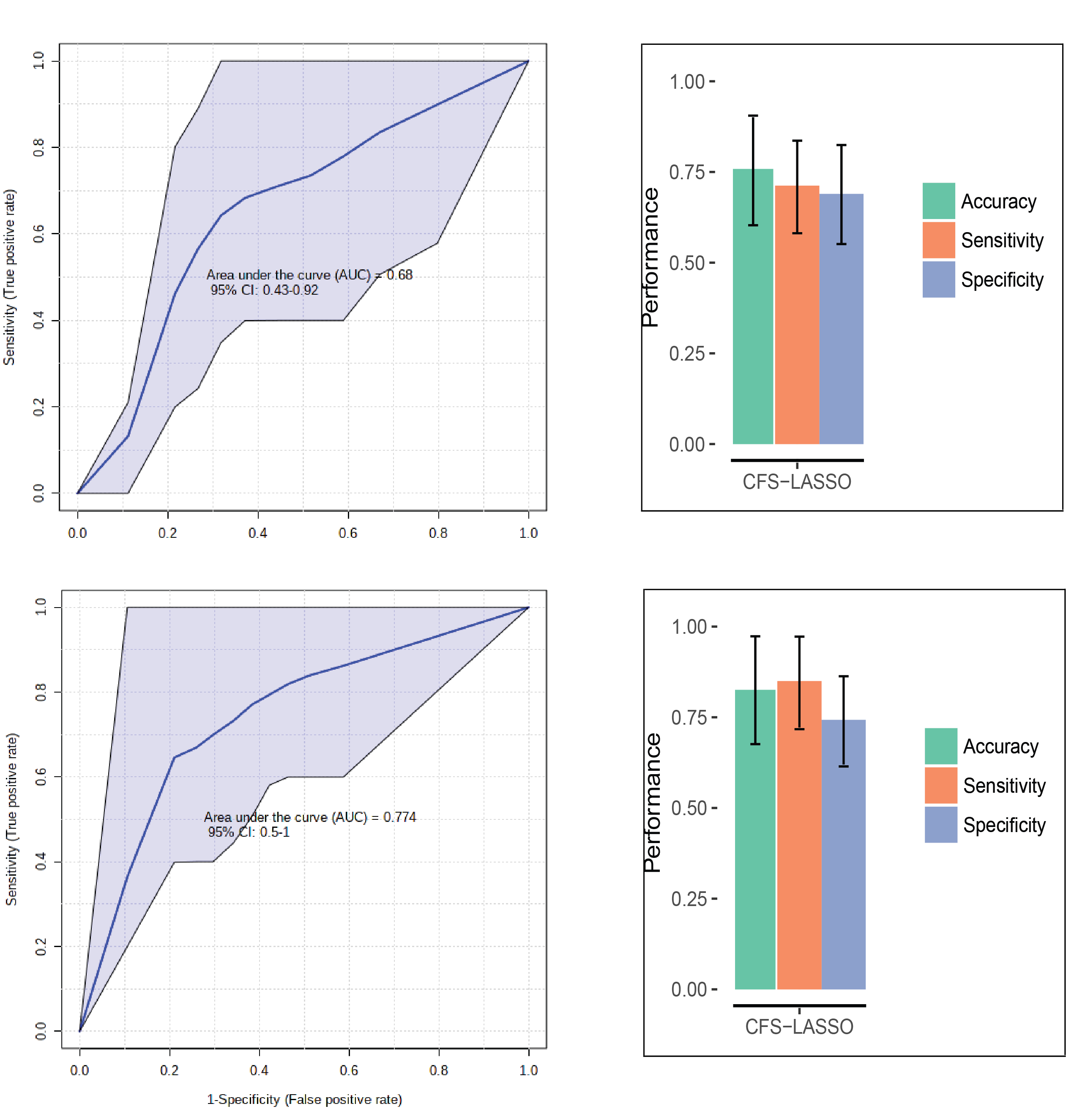

Supplement: Supplementary file 1 [file metabolites-10-00261-s001.zip › supplementary/Supplementary Figure 3.png]
